# Stimulatory effect of fluoxetine and desipramine, but not mirtazapine on C26 colon carcinoma hepatic metastases formation: association with cytokines

**DOI:** 10.3389/fimmu.2023.1160977

**Published:** 2023-06-20

**Authors:** Marta Kubera, Beatriz Arteta, Beata Grygier, Katarzyna Curzytek, Stanisław Malicki, Michael Maes

**Affiliations:** ^1^ Department of Experimental Neuroendocrinology, Maj Institute of Pharmacology Polish Academy of Sciences, Krakow, Poland; ^2^ Department of Cell Biology and Histology, School of Medicine and Nursing, Tumor Microenvironment Group, Basque Country University, Leioa, Spain; ^3^ Department of Microbiology, Faculty of Biochemistry, Biophysics and Biotechnology, Jagiellonian University, Krakow, Poland; ^4^ Malopolska Centre of Biotechnology, Jagiellonian University, Krakow, Poland; ^5^ Department of Psychiatry, Faculty of Medicine, King Chulalongkorn Memorial Hospital, Bangkok, Thailand; ^6^ Department of Psychiatry, Medical University of Plovdiv, Plovdiv, Bulgaria; ^7^ IMPACT Strategic Research Centre, Deakin University, Geelong, VIC, Australia; ^8^ Kyung Hee University, Seoul, Republic of Korea

**Keywords:** antidepressants, desipramine, fluoxetine, mirtazapine, C26 colon carcinoma, cytokines

## Abstract

Due to the high prevalence of depression among cancer patients, antidepressant medications are frequently administered as adjuvant treatment. However, the safety of such medications in the development of metastasis is unclear. In this study, we investigated the effects of fluoxetine, desipramine, and mirtazapine on the liver metastasis of murine C26 colon carcinoma (cc). Balb/c male mice were administered these antidepressants intraperitoneally (i.p.) for 14 days following intrasplenic injections of C26 colon carcinoma cells. Desipramine and fluoxetine, but not mirtazapine, significantly increased the number of tumor foci and total volume of the tumor in liver tissue. This effect was associated with a decrease in the ability of splenocytes to produce interleukin (IL)-1β and interferon (IFN)-γ and an increase in their ability to produce interleukin (IL)-10. Similar changes were observed in plasma IL-1β, IFN-γ, and IL-10 levels. The current study demonstrates that the stimulatory effect of desipramine and fluoxetine, but not mirtazapine, on experimental colon cancer liver metastasis is associated with a suppression of immune defenses against the tumor.

## 1 Introduction

Effective drug therapy for depression is important for patients with cancer, considering that the quality of life is often destroyed because of the stress connected with the fear of illness progression, loss of independence and dignity, the side effects of surgery, radio- and/or chemotherapy, and fear of death. The recently published study shows that antidepressants such as selective serotonin reuptake inhibitors (SSRIs) and mirtazapine, were effective and well tolerated in children and adolescents with cancer and psychiatric comorbidities (1).

Some reports have suggested that antidepressants may be linked with the formation and growth of tumors, raising concern about prescribing such medication on a regular basis (2–5). On the contrary, some preclinical studies showed no carcinogenesis in mice receiving a human-equivalent dose of antidepressant drugs for 24 months (4), as well as some clinical systematic rereviews and dose-response meta-analyses found that antidepressant use did not increase the incidence risk of cancers and even decreased the incidence risk of breast cancer, along with a non-linear or linear dose-response relationship (6). Therefore, the effects of these antidepressant drugs on tumors remain unclear.

There is also evidence that antidepressants have negative immunoregulatory and anti-inflammatory effects (7–10). Thus, in human depression and animal models of depression antidepressants, such as tricyclics and SSRIs, suppress the production of interferon (IFN)-γ and interleukin (IL)-1β and increase the production of IL-10, a negative immunoregulatory cytokine. These immunoregulatory effects may play a role in tumor progression or metastasis processes.

Fluoxetine hydrochloride, an SSRI, blocks the reuptake of serotonin into the presynaptic nerve terminals, resulting in enhanced synaptic serotonin levels. It is one of the most commonly used antidepressants, due to its efficacy, safety, and tolerability. The SSRIs citalopram, escitalopram, sertraline, and fluoxetine are generally the first-line choice for the treatment of depression, bulimia nervosa, and obsessive-compulsive disorder. Desipramine displays an antidepressant property similar to that of other tricyclic antidepressants (TCAs). It is the active “*in vivo*” metabolite of imipramine and as such, shares many of imipramine’s pharmacologic effects. It is not considered a first-line treatment since the introduction of SSRIs. Desipramine inhibits the reuptake of noradrenaline much stronger than serotonin. Mirtazapine is an effective new-generation antidepressant, which enhances noradrenergic neurotransmission *via* an antagonistic action at α_2_-adrenergic auto- and heteroreceptors. Mirtazapine has been suggested as a first choice during cancer immunotherapy because of its potent anti-nausea effects, which stimulate appetite in cancer patients (11, 12).

The aim of this work was to evaluate the effect of chronic treatment with three antidepressants representing various pharmacological groups: desipramine, fluoxetine, and mirtazapine on the metastatic potential of murine C26 colon carcinoma cells to the liver. Colorectal cancer is one of the most aggressive cancers in Western countries. Patients suffering from this cancer die from metastases, mainly to the liver, rather than from the primary tumor. Hence, we examined the effect of repeated administration of antidepressants on the development of metastatic foci in the liver. Furthermore, to better understand the association between antidepressant effects on tumor pathology and immune system activity, the T-cell proliferative response to concanavalin A (Con A), and the production of anti- and pro-tumoral cytokines: IL-1β, IL-10, and IFN-γ were also studied.

## 2 Materials and methods

### 2.1 Animals

The experiment was performed in the Basque Country University School of Medicine & Nursing on 75 animals (6–8 weeks old male Balb/c mice) obtained from Charles River, Barcelona, Spain. Animals were housed in up to five per cage, at 22°C and 40% humidity under a 12-hour light-dark cycle, with free access to water and standard food. All the proceedings were approved by the Basque Country University Ethical Committee (CEID) and by institutional, national, and international guidelines regarding the protection and care of animals used for scientific purposes.

### 2.2 Cell culture

Murine C26 colon carcinoma cell line is syngeneic to Balb/c mice (13). C26 cc cell line was obtained from ATCC (Manassas, VA, USA) and was cultured in culture flasks filled with RPMI-1640 supplemented with 10% Fetal Calf Serum, penicillin, and streptomycin. The cell cultures were maintained at 37°C in 5% CO_2_. Cells in the exponential growth phase were harvested at 80% confluence, the cell suspension was centrifuged, washed once with phosphate-buffered saline (PBS), and resuspended in PBS at a concentration of 2 × 10^6^ cells per mL. Cell viability was determined by trypan blue exclusion. Only single-cell suspensions with 95% viability were used for inoculation.

### 2.3 *In vivo* experimental development of hepatic metastases

Animals were divided into five experimental groups consisting in five experimental units each. The experiment was repeated three times given a total of N=75, n=15, and of N=25, n=5 per repetition. To induce hepatic metastases *in vivo*, C26 cc cells were suspended at a concentration of 1.5 × 10^5^ in 0.1 ml of Hanks’ Balanced salt solution (HBBS) and were intrasplenically (i.s.) injected into anesthetized mice (Nembutal, 50 mg/kg) as previously described (14). Mice were anesthetized for portal blood collection, and then sacrificed by cervical dislocation on the 15^th^ day after the injection of cancer cells and livers were removed. Livers were fixed by immersion in Zinc solution for 12 hours at room temperature, and then, paraffin-embedded. A minimum of six 5-μm thick tissue sections of the liver (three groups, separated 1 mm) were stained with hematoxylin and eosin (H-E). An integrated image analysis system (Olympus Microimage 4.0 capture kit) connected to an Olympus BX51TF microscope was used to quantify the number, average diameter, and position coordinates of metastases. The percentage of the liver volume occupied by metastatic tissue and metastases density (foci number/100 mm^3^) were also determined (15). In the case of outliers, animals dying before the established endpoint, with ascites and/or inflammation, and/or little or no sign of growth in the spleen and/or liver will not be considered for metastatic quantification.

### 2.4 Antidepressant drugs treatment

Balb/c mice were subdivided into 5 groups of 5 animals each and received daily intraperitoneal (i.p.) injections of either desipramine (10 mg/kg, Research Biochemicals International, USA), fluoxetine (10 mg/kg, The Lilly Laboratories, USA), or mirtazapine (20 mg/kg, Organon, The Netherlands) for 14 days, respectively (16, 17). Desipramine and fluoxetine were dissolved in 0.9% saline, whereas mirtazapine was suspended in a 1% aqueous solution of Tween 80. The Control group for desipramine and fluoxetine groups received i.p. injection of saline (NaCl group), whereas the control group for mirtazapine studies received 1% aqueous solution of Tween 80 (Tween group). The first drug injection was given 2 h after injection of tumor cells, and the last drug injection 24 hours before sacrificing the animals. The experiments were repeated three times.

### 2.5 Proliferative response of splenocytes to mitogen stimulation *in vitro*

Spleens from tumor-bearing mice were aseptically removed, minced in PBS-EDTA (1% w/v) using a pair of scissors, and passed through fine steel (70 μm diameter) mesh to obtain a homogeneous cell suspension. After centrifugation (380 × g at 4°C for 10 min), the pelleted cells were washed three times in PBS and resuspended in RPMI 1640 complete medium. Splenocytes were cultured for 72 h at a concentration of 4 × 10^6^ cells/ml in the presence or absence of 2.5 μg/ml Con A. Proliferation was quantified utilizing 3-(4,5-dimethylthiazol-2-yl)-2,5-diphenyltetrazolium bromide (MTT, Sigma) assay and the absorbance was evaluated after 4 hours in an ELISA reader (Bio-Rad, USA) at 570 nm (18).

### 2.6 Determination of cytokines

Previously collected blood from tumor-bearing mice was centrifuged and serum was collected and stored at -80°C for analysis of cytokines later. Splenocyte suspensions were seeded at a concentration of 4 × 10^6^ cells/ml in 24-well Corning tissue culture plates and were then stimulated with Con A solution (2.5 µg/ml) and LPS solution (5 µg/ml) simultaneously or remained unstimulated. Cell-free supernatants were collected 48 h later and stored at -20°C until use. IL-1β, IL-10, and IFN-γ levels were estimated in serum and supernatants. All the enzyme-linked immunosorbent assays (ELISA) were based on monoclonal-monoclonal antibody pairs and were performed using DuoSet ELISA Development Kits (R&D Systems, Abingdon, UK) following manufacturer instructions.

### 2.7 Statistical analysis

For statistical analysis of the results, Statistica 9.0 software (Statsoft, Tulsa, USA), run on a PC-compatible computer, was used. Intraexperimental statistical significance was evaluated by a two-tailed unpaired t-Test and the differences between groups were considered statistically significant when p<0.05. Then, differences between independent experiments were evaluated using a one-way analysis of variance (ANOVA) followed by Dunnett's test. The normality of variable distributions and homogeneity of variances were verified by the Shapiro-Wilk and Levene’s tests, respectively. The values that exceeded 2-fold the standard deviation from the average were discarded from the analyses. The p-values lower than 0.05 were regarded as statistically significant.

## 3 Results

### 3.1 The effect of antidepressant drugs on C26 colon carcinoma liver metastasis development

**Figure 1:**
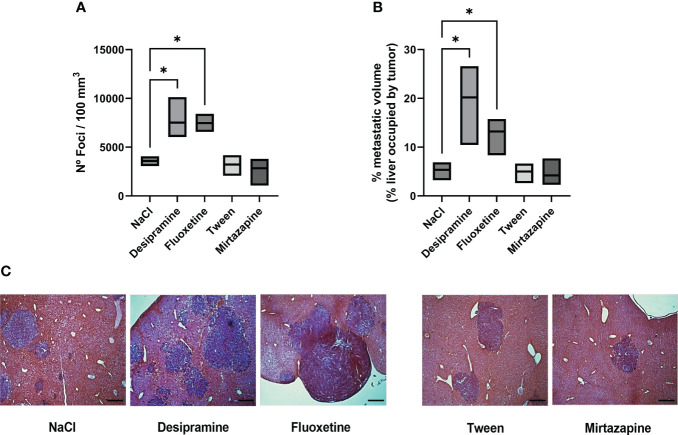
Effect of antidepressants on C26 colon carcinoma (cc) metastatic colonization to the liver. Balb/c mice were injected intrasplenically (i.s.) with 1.5 × 10^5^ C26 colon carcinoma cells. Animals were treated intraperitoneally (i.p.) with a daily dose of desipramine (10 mg/kg), fluoxetine (10 mg/kg), mirtazapine (20 mg/kg), or proper vehicle (0.9% NaCl or 1% Tween) for 14 days. The first drug injection was given 2 h after intrasplenic injection of tumor cells. After 15 days mice were sacrificed and livers were processed for histology. A series of three experiments (5 mice per treatment) was carried out to determine metastatic growth in the liver; the number of foci per 100 mm^3^ of liver tissue **(A)**, metastatic volume (overall tumor tissue volume of liver occupied by metastasis) **(B)**, microphotography of H-E staining of livers collected from tumor-bearing mice after antidepressant treatment, bar = 250 µm **(C)**. Data represent the minimum, maximum and mean values of three micro experiments, the effects of the different treatments on the rate of metastatic development were compared by one-way analysis of variance (ANOVA), *p<0.05.

In order to analyze the effect of antidepressant drugs on the metastatic potential of C26 cc cells to the liver, tumor cells were injected i.s. as described in the “Materials and methods” section. The animals were treated with a daily dose of either antidepressant or vehicle until the day before sacrifice 15 days after tumor cell injection. **Figure 1** shows that both desipramine and fluoxetine significantly increased the number of C26 cc cell foci and the overall metastatic volume estimated as the percentage of liver tissue occupied by the tumor when compared to that tissue from vehicle-treated control mice. On the contrary, mirtazapine did not modulate the number and volume of metastases in comparison to vehicle-treated control mice.

### 3.2 Production of cytokines

**Figure 2:**
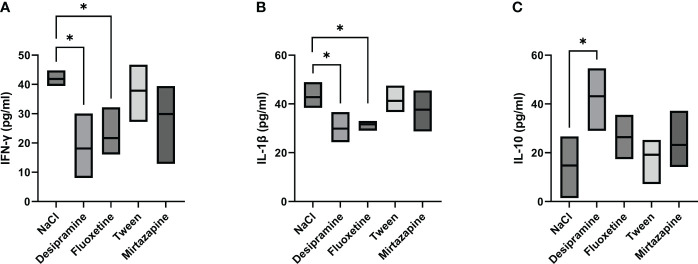
Effect of antidepressants on cytokine levels in serum from C26 colon carcinoma-bearing mice. Balb/c mice were injected i.s. with 1.5 × 10^5^ C26 colon carcinoma cells. Animals were treated i.p. with a daily dose of desipramine (10 mg/kg), fluoxetine (10 mg/kg), mirtazapine (20 mg/kg), or proper vehicle (0.9% NaCl or 1% Tween). After 15 days mice were sacrificed and serum was collected for cytokine analysis. A series of three experiments (5 mice per treatment) was carried out to determine levels of IFN-γ **(A)**, IL-1 β **(B)**, and IL-10 **(C)**. Data represent the minimum, maximum and mean of three micro experiments. The effects of the different treatments on cytokine levels were compared by one-way analysis of variance (ANOVA), *p<0.05.

**Figure 3:**
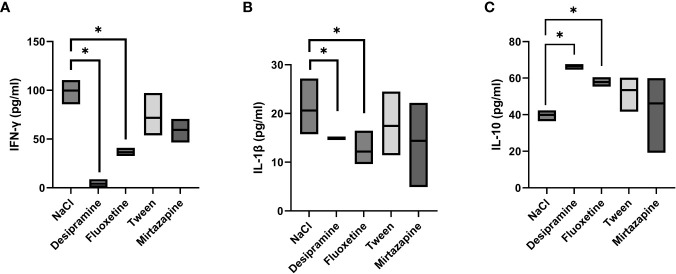
Effect of antidepressants on cytokine levels produced by spleen lymphocytes from C26 colon carcinoma-bearing mice. Balb/c mice were injected i.s. with 1.5 × 10^5^ C26 colon carcinoma cells. Animals were treated i.p. with a daily dose of desipramine (10 mg/kg), fluoxetine (10 mg/kg), mirtazapine (20 mg/kg), or proper vehicle (0.9% NaCl or 1% Tween). After 15 days mice were sacrificed and spleens were collected for cytokine analysis. After isolation, spleen lymphocytes were cultured for 48 h in presence of Con A and LPS simultaneously stimulation. A series of three experiments (5 mice per treatment) was carried out to determine levels of IFN-γ **(A)**, IL-1β **(B)**, and IL-10 **(C)**. Data represent the minimum, maximum and mean of three micro experiments. The effects of the different treatments on cytokine levels were compared by one-way analysis of variance (ANOVA), *p<0.05.

Next, the levels of serum inflammatory cytokines involved in the immune response developed during metastatic development were analyzed. **Figures 2A, B**, **3A, B** show that the levels of both serum and spleen IFN-γ and IL-1β were decreased after desipramine- and fluoxetine- but not those of mirtazapine in C26 cc cells-bearing mice after 14 days of treatment. Particularly drastic was the decrease in spleen IFN-γ levels in desipramine-treated tumor-bearing mice (up to 96%, **Figure 3A**). Desipramine significantly increased (214%) serum IL-10 levels (**Figure 2C**), whilst desipramine and fluoxetine increased the levels of this cytokine produced by the splenocytes collected from tumor-bearing mice (**Figure 3C**).

### 3.3 Proliferative activity of splenocytes of tumors bearing mice

**Figure 4:**
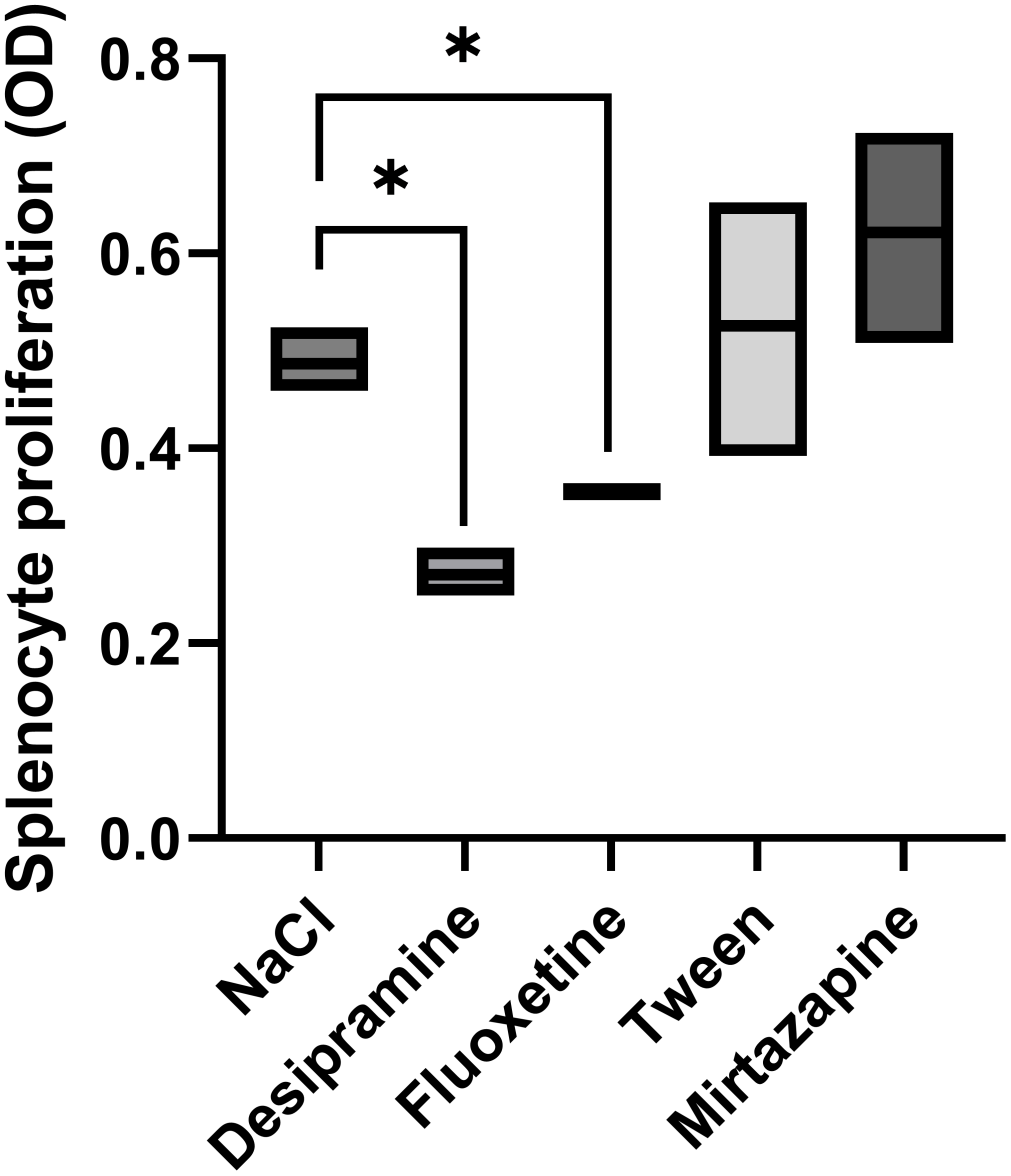
Effect of antidepressants on spleen lymphocyte proliferation. Animals were treated i.p. with a daily dose of desipramine (10 mg/kg), fluoxetine (10 mg/kg), mirtazapine (20 mg/kg), or proper vehicle (0.9% NaCl or 1% Tween). Spleen lymphocytes were isolated from spleens collected from C26 colon carcinoma-bearing mice 15 days after tumor cell injection. After isolation, spleen lymphocytes were cultured for 72 h in the presence of Con A, and the proliferation of lymphocytes was measured by MTT assay. Data represent min, max and mean of three micro experiments. The effects of the different treatments on splenocyte proliferation levels were compared by one-way analysis of variance (ANOVA), *p<0.05.

The proliferation of splenocytes is a key factor in antitumor functions. Thus, we analyzed the proliferative activity of splenocytes in tumor-bearing mice treated with an antidepressant. Desipramine and fluoxetine significantly inhibited the proliferative activity of splenocytes isolated from C26 cc cell-bearing mice by 36% and 28% respectively. Conversely, the blastogenic response of splenocytes to Con A was not changed in mirtazapine-treated Balb/c mice with C26 cc metastasis in the liver (**Figure 4**).

## 4 Discussion

Antidepressants are used in cancer patients, but the effects of antidepressant drugs on the progression of cancer metastasis are not fully elucidated. In the present study, we aimed to put some light by analyzing the effects of several antidepressants in the metastatic progression of murine C26 colon carcinoma to the liver. The main findings of this study are that treatment of C26 colon carcinoma cells-bearing mice with desipramine or fluoxetine: 1) increases the metastatic potential of C26 cc cells to the liver; 2) inhibits the proliferative activity of splenocytes; and 3) decreases the production of pro-inflammatory cytokines (IFN-γ, IL-1β) whilst increasing the production of the anti-inflammatory cytokine IL-10. Mirtazapine, on the other hand, had no effect on colon carcinoma growth in the liver as well as on immune system activity.

To the best of our knowledge, the effect of antidepressant drugs on C26 cc tumor progress in a unique model of liver metastasis formation after intra-splenic tumor cells injection has not been studied yet. The intra-splenic injection of tumor cells offers advantages over other murine models, such as consistent metastatic development to the liver at a uniform time, peripheral blood immune analysis, and high reproducibility. Also, tumor cells are allowed to reach the liver through their natural way, that is, through the portal vein (19). Previous studies showed that antidepressant drugs given after injection of tumor cells inhibit jejunal and colonic tumors in rats and xenografts of human colorectal and prostate cancers in athymic nude mice (20) or enhance tumor growth. Brandes et al. (4) found that fluoxetine stimulated the development of chemically-induced tumors in rodents but the doses were much higher than those used in our study. Fluoxetine in a dose of 20-80 mg/kg per day decreases the latency of C-3 fibrosarcoma and aggregates the weight of melanoma and the rate and frequency of development of mammary tumors in rats fed with dimethylbenzanthracene. The prometastatic effect of fluoxetine has been used in invasive and metastatic models in human HepG2 cells (21). The levels of serotonin, which are increased by SSRIs, may promote tumor growth in human hepatocellular cancer (22).

Moreover, fluoxetine (below 25 µM) stimulates the accumulation of fat in primary hepatocytes, causing the liver to become fatty, favoring a profibrotic environment which may, in turn, contribute to the development of liver sclerosis and liver cancer (23). Moreover, fluoxetine treatment may increase the number of breast cancer metastases to the brain in a mouse model (24). Contrary to the above-mentioned data several reports revealed a beneficial, anti-tumor effect of fluoxetine. Treatment of fluoxetine significantly decreased the progression of hepatocellular carcinoma Hep3B and non-small cell lung cancer CL1-5-F4 tumors (25). Similar anti-tumor and anti-metastatic properties have been reported for imipramine, related to the dose-dependent inhibition of Fascin-1, the overexpression of which is correlated with the progression of colorectal tumors (26). Furthermore, a recent retrospective cohort study revealed that individuals prescribed fluoxetine and other SSRIs had a reduced risk of bladder cancer (27). The differences observed in tumor development might be explained by differences in the tumor microenvironment affected by the progression of cancer. In fact, the phenotypic and genotypic characteristics of tumor cells from a primary tumor might differ greatly from those observed in their metastatic counterparts. Under such conditions, the immune microenvironment may also adapt to the new scenario. In addition, the present model focuses on the later steps of the metastatic process, namely, adhesion to secondary organ endothelium, transmigration to the organ parenchyma, and final colonization, including the avascular and vascular phases. Further studies should put some light on the effects of these anti-depressants in earlier metastatic steps, such as primary tumor detachment, intravasation, and pre-metastatic niche formation.

In our previous studies, we observed that chronic desipramine and fluoxetine treatment, administered before the injection of tumor cells, induced gastrointestinal tract, peritoneal cavity, skin, and spleen metastasis formation by B16F10 melanoma cells in C57BL/6J male and female mice (7, 28). The effect was age and “temperament”-dependent. Desipramine pretreatment dramatically promoted metastasis formation and increased mortality rate in young high-active males whereas fluoxetine pretreatment induced such effect in young high-active females (28). In our former studies in the lung metastasis cancer model induced by i.v. injection of MADB 106 cells to Wistar rats subjected to stress procedure according to the chronic mild stress (CMS) model of depression, pro-metastatic effects caused by desipramine treatment were observed. These studies showed a very disadvantageous effect of desipramine on tumor progression in the lungs of both stress highly-sensitive and stress non-reactive (stress resistant) animals (29). Moreover, desipramine injection decreased the number of T CD8+ cells and cytotoxicity of natural killer (NK) cells as well as skew macrophage activity towards M2 functional phenotype (30).

The development of cancer metastases is particularly affected by the inhibition of immune activity associated with immune checkpoints (ICP), especially the PD1 (programmed death receptor 1) present, among others, on CD8+ T cells, natural killer cells and macrophages, and its ligand PD-L1 (programmed death receptor ligand 1) presented on cancer cells (31, 32). Our primary results show a reduction in PD-L1 expression in the brain tissue of two different species of animals in two animal models of depression (Curzytek K, Malicki S, Kubera MW, Maes M, et al., paper in preparation). This observation confirms our hypothesis that depressive disorders may correlate with ICPs dysregulation, both in the nervous and immune systems. It suggested a significant impact of antidepressants on the expression of PD-1 and/or PDL-1 on cells of the immune system and cancer cells (increased expression of PD-1 on cells eliminating cancer cells and, at the same time, increased expression of PD-L1 on cancer cells, induced by some antidepressants, may be an important cause of the progression of the neoplastic process).

The third drug evaluated in this study – mirtazapine – did not show any effect on either metastases formation or the tested immune parameters. Mirtazapine appears to be a promising drug in the alleviation of cancer-related depression symptoms, although more randomized controlled trials are needed to define its effectiveness and safety in the cancer population (33).

The promotion of metastasis formation in animals treated with fluoxetine and desipramine is probably connected with the inhibition of anti-cancer cell-mediated immunity. In the present study, we have shown that fluoxetine and desipramine decrease Con A-induced splenocyte proliferation. Moreover, this was accompanied by a decrease in the ability of splenocytes to produce IL-1β and IFN-γ, and by an increase in IL-10 production and similar changes in plasma cytokine levels. However, our former studies were shown that simvastatin (used to decrease the high level of blood cholesterol) although exhibits pleiotropic effects on carcinogenesis, inhibits the release of IL-8 and IL-6 from colorectal cell lines and decreases serum IL-6 level in patients with advanced colorectal cancer (34).

In the present study, we observed a reduction in IL-1β levels after desipramine and fluoxetine treatment which correlates with an increase in the metastatic burden observed in the liver. This is in contrast with other several studies showing that IL-1β produced by tumor-associated macrophages supports colon cancer cell growth (35) and stimulates the adhesion of colon carcinoma cells to mesothelial monolayers (36) and lung microvascular endothelium (37). Moreover, it was shown that sialyl Lewis X-expressing antigens on colon cancer cells act as ligands for selectins, induced by IL-1β, on human liver endothelial cells (38). On the other hand IL-18, a cytokine with strong antitumor activity and an IFN-γ-inducing factor in experimental models requires processing by an IL-1 converting enzyme (39). Therefore, we may speculate that a decrease of IL-18 may also occur in antidepressant-treated C26 cc metastasis-bearing Balb/c mice, avoiding the mounting of an efficient immune response in the liver. In addition, a decrease of IL-1β, *via* a reduction in the production of IL-2 in T spleen cells, may be responsible for the decreased proliferative activity of splenocytes. Also, the involvement of both pro-inflammatory cytokines in the development of an adequate angiogenic vascular might suggests that the effect of antidepressants examined in this study is independent of this pathway, and relates in a unique way to the local immune response developed in the liver during metastatic progression of colorectal cancer.

Bevers et al. (2022) showed a significant role in the activation of dendritic cells, tumor-specific T CD8+ cells, and macrophages in the spleen and an increase in the production of Type I interferons by splenocytes in the formation of an anti-tumor response in C57BL/6J mice (40).

Maybe in our study desipramine and fluoxetine inhibits the spleen and liver formation of long-lasting tumor-specific T CD8+ cells, which showed a high degree of multifunctionality and provoked high antitumor efficiency.

IFN-γ is a multifunctional cytokine that plays important role in activating innate and adaptive immune response *via* enhancing NK cells, T cells, and natural killer T (NKT) cells (41). The decrease of IFN-γ production predisposes to the development of primary and transplanted tumors, whereas increases promote host response to tumors, although the mechanisms explaining these effects of cytokines have remained elusive (42). It was shown that IFN-γ has antiproliferative effects on pancreatic cancer cells and inhibits pancreatic tumor growth in Balb/c nu/nu mice (43) and experimentally induced skin carcinogenesis (44).

Conversely, IL-10 levels play an important role in the modulation of tumor progression: high levels of IL-10 in a tumor microenvironment block the activity of antigen-presenting cells (APC), cytotoxic T cells, and NK cells and stimulate tumor growth. We observed that the higher metastatic growth in C26 cc cell-bearing mice after desipramine and fluoxetine treatment may be associated with an increased ability of splenocytes to produce IL-10. This mechanism may decrease anti-tumor immunity and enhance metastasis by promoting the ability to circulate C26 cc cells to survive in the host liver. This is in accordance with the increase in IL-10 levels in soluble Intercellular Adhesion Molecule 1 (sICAM-1)-induced hepatic metastasis formation (14). IL-10 may also enhance weakly metastatic human colorectal carcinoma metastasis into nude mouse liver *via* inhibition of the production of nitric oxide (NO), reactive oxygen species (ROS), and upregulation of expression of inducible nitric oxide synthetase (iNOS) in the host liver (29, 45).

A bioinformatics approach identified antitumor effects of tricyclic antidepressants in small cell lung cancer (SCLC) and other high-grade neuroendocrine carcinomas (grade 3 neuroendocrine carcinomas, G3NEC) that were subsequently validated in preclinical models (46). Unfortunately, our research shows that desipramine increases the formation of metastases to the liver and in this situation, we cannot recommend repositioning desipramine in clinical trials in colorectal cancer.

In conclusion, the present study showed prometastatic effects of desipramine and fluoxetine, but not mirtazapine, by modulating secondary liver colonization during colon carcinoma progression, and that these effects are associated with the effects of antidepressants on cytokines, i.e., enhanced IL-10 and reduced IL-1 β and IFN-γ levels.

## Data availability statement

The raw data supporting the conclusions of this article will be made available by the authors, without undue reservation.

## Ethics statement

The animal study was reviewed and approved by Basque Country University Ethical Committee (CEID).

## Author contributions

Conceptualization, MK and BA; methodology, BA and MK; formal analysis, BA, BG, and KC; investigation, BA; resources BA and MK; writing – original draft preparation, MK and BA; writing – review and editing, BA, BG, KC, MK, SM, and MM; supervision, MK; project administration, MK; funding acquisition, MK. All authors contributed to the article and approved the submitted version.
